# Workplace Factors That Affect Medical Educator Well-being: Insights From A National Survey of Osteopathic Medical Faculty

**DOI:** 10.1007/s40670-025-02586-3

**Published:** 2025-11-28

**Authors:** Zachary M. Himmelberger, Brooke Johnson, Kathryn Carey, Yoi Tibbetts, Kenneth E. Barron, Chris S. Hulleman, Mark R. Speicher

**Affiliations:** 1 Motivate Lab, Harrisonburg, VA USA; 2 OMS-IV, A.T. Still University Kirksville College of Osteopathic Medicine, Kirksville, MO USA; 3 University of Virginia, Charlottesville, VA USA; 4 James Madison University, Harrisonburg, VA USA; 5 American Association of Colleges of Osteopathic Medicine, Bethesda, MD USA

## Abstract

Despite a recent focus on healthcare student and worker well-being, few studies have investigated the correlates of medical educator well-being. To address this limitation, this study examined workplace factors associated with burnout, job satisfaction, and likelihood to recommend the workplace among medical educators at 42 osteopathic medical campuses in the U.S. Our sample included 466 educators (47% female, 11% underrepresented in medicine [i.e., black, Hispanic/Latino, Native American, Pacific Islander], 59% clinical) who completed measures of perceived professional development support, workplace belonging, workplace inclusivity, work-life balance, burnout, job satisfaction, and likelihood to recommend their workplace. Results indicated that perceived professional development support, workplace belonging, workplace inclusivity, and work-life balance were all significantly associated with our outcomes: burnout, job satisfaction, and likelihood to recommend their workplace. Regression analyses revealed that these workplace factors, together with personal identities (i.e., gender, race/ethnicity) and academic discipline, explained a significant proportion of variance in all three outcomes. We also found significant differences in both the predictor and outcome variables based on gender, race/ethnicity, and academic discipline (i.e., basic science, clinical) highlighting significant disparities in educator experiences based on their personal identities and discipline. Targeted institutional initiatives promoting professional development, workplace belonging, inclusivity, and work-life balance may increase job satisfaction, decrease burnout, and address workplace disparities.

The global pandemic intensified an already growing crisis in healthcare, drawing urgent attention to rising rates of burnout and turnover among healthcare workers, particularly in clinical settings. However, less attention has been paid to the well-being of medical educators, some of whom are also practicing physicians. Medical educator well-being is critical because these faculty serve as role models for medical students, many of whom are experiencing high rates of burnout and depression themselves [1–3]. Additionally, replacing faculty who leave due to burnout or dissatisfaction is costly and disrupts the continuity of medical education programs [4]. In the current study, we explore how workplace factors predict burnout and job satisfaction in medical school faculty, as well as describe differences between medical educators based on their personal identities (i.e., gender and race/ethnicity) and academic discipline (i.e., basic science or clinical).

Research has identified that medical educators have disparate experiences in the workplace based on their personal identities. For example, women report more burnout, lower belonging, and less gender equity [5, 6]. Although the mechanisms behind gender differences in burnout are not fully understood, evidence from clinical settings suggests that female physicians face unique challenges, including “a higher workload, spending more time in the electronic health records, and more time per patient,” as well as having access to fewer resources compared to their male counterparts [7]. Broader societal expectations and pressures may also contribute to these disparate work experiences [8]. Furthermore, medical educators part of an underrepresented racial or ethnic group (i.e., black, Hispanic/Latino, Native American, Pacific Islander) experience less belonging, perceive the workplace as less inclusive, and more than 20% report experiencing racial/ethnic discrimination [9]. These educators also face unique stressors that impact their workplace experience such as difficulty of cross-cultural relationships, different performance expectations, and the responsibility of being a leader in diversity efforts [10]. Taken together, there is strong evidence that women and medical educators from underrepresented racial or ethnic backgrounds have disparate workplace experiences that may lead to increased burnout and reduced job satisfaction.

Studies have also shown that nearly half of U.S. physicians report at least one symptom of burnout, which is comparable to clinical academic faculty at Liaison Committee on Medical Education accredited institutions [11–13]. However, few studies have investigated burnout among basic science medical educators. In one large-scale study conducted by the Association of American Medical Colleges, the prevalence of burnout was higher for faculty in clinical departments who provided patient care (31%) compared to faculty in clinical departments who do not provide patient care (28%) or faculty in basic science departments (26%) [14]. This finding was consistent with a study in Iran that found clinical faculty showed higher burnout than their basic science colleagues [15].

 To the authors’ knowledge, there have been no large-scale investigations of osteopathic medical school faculty burnout or job satisfaction. There are systematic differences between allopathic and osteopathic medical schools that could affect faculty well-being. For instance, osteopathic medical schools use a more community-based, distributed model of clinical education, tend to be in more rural settings, the clinical faculty may be more likely to have a D.O. degree themselves, and there has been a recent and on-going expansion in the number of osteopathic medical schools, so these faculty may be experiencing more transitions as new cohorts of students begin. Relatedly, there are also significant differences between osteopathic medical schools based on location, size, and finances, which may affect the available resources to support faculty well-being. 

Although few studies focus specifically on medical educators, related research shows that workplace factors such as professional development support and a sense of belonging are associated with decreased burnout and increased job satisfaction. This issue holds particular importance because medical educators who experience burnout often report lower job satisfaction and a stronger intent to leave the profession [14]. In a recent literature review, Shiri et al. found that professionals who have access to development opportunities are more likely to stay in their roles [16], and those who view these opportunities positively tend to report greater job satisfaction [17]. Furthermore, organizational belonging, which reflects feeling valued and included by one’s organization and colleagues, also plays a key role. Medical students who report a strong sense of belonging tend to flourish and show few signs of burnout [18]. Researchers have identified similar patterns in recent nursing graduates and educators [18–20]. Among university faculty, those who feel supported by their department and valued by their peers also report lower levels of burnout [21]. In summary, a growing body of evidence highlights how workplace conditions shape medical educator well-being and satisfaction. However, researchers still need to examine how these dynamics affect medical educators and whether they vary based on personal identities. 

In the present study, we conducted a comprehensive investigation of workplace experiences among medical educators in osteopathic medical schools. We analyzed data from a large, national survey conducted across 42 campuses at colleges of osteopathic medicine in the United States. This exploratory study examined a broad set of workplace predictors (e.g., perceived professional development support, workplace belonging, workplace inclusivity, and work-life balance) and their relationships with key outcomes (e.g. burnout, job satisfaction, and likelihood of recommending the workplace). Our research adds to the existing literature by extending findings among allopathic medical educators to their osteopathic peers and from clinical faculty to basic science faculty. Further, we aim to expand research from other professions supporting the relationship between workplace factors and burnout to medical educators. We hope our findings can inform future efforts, by researchers and practitioners, to support the well-being and retention of medical educators.

## Materials and Methods

### Study Design

This study employed a cross-sectional survey design as part of a broader faculty development initiative. In January 2022, the American Association of Colleges of Osteopathic Medicine (AACOM) was awarded a 3-year federal grant to provide professional development for medical educators with the aim of promoting wellness and resiliency in future physicians. From September 1 st to October 20th of 2023, AACOM administered a national survey of faculty at 42 osteopathic medical school campuses in the United States. The survey included items selected by or developed by experts at AACOM and Motivate Lab, an educational research lab, including the authors MS, YT, and ZH, and experts from the Society for Osteopathic Medical Educators and the National Academy of Osteopathic Medical Educators. As a part of the broader survey, we assessed professional development needs and preferences, factors associated with workplace satisfaction, and well-being. In the present study, we report on measures of perceived professional development support, workplace belonging, workplace inclusivity, work-life balance, burnout, job satisfaction, and likelihood to recommend their workplace.

### Participants and Inclusion Criteria

A total of 688 educators opened the survey link. Respondents who did not complete any of the seven core measures (i.e., perceived professional development support, workplace belonging, workplace inclusivity, work-life balance, burnout, job satisfaction, and likelihood to recommend their workplace) were excluded from analysis (*n* = 222). Due to small sample sizes in some races or ethnicities, medical educators were grouped URiM (American Indian or Alaskan Native, Black or African American, Hispanic/Latino) or non-URiM (Asian, non-Hispanic, White). We also grouped medical educators as clinical or basic science faculty based on their department or specialty.

### Instruments and Measures

Perceived professional development support, workplace belonging, workplace inclusivity, and work-life balance were assessed with scales developed for the purposes of this survey. Perceived professional development support was measured with four items (𝛼 = 0.93) examining faculty perceptions of institutional support, values, and resources provided by their institution for professional development in teaching. The workplace belonging scale consisted of three items (𝛼 = 0.91) evaluating the extent to which faculty feel accepted, valued, and included in their workplace. Workplace inclusivity was measured using three items (𝛼 = 0.66) to assess the extent to which faculty felt their identity influenced colleagues’ perception of them and the extent to which discrimination is dealt with appropriately. The work-life balance scale consisted of two items (𝛼 = 0.78) that examined the extent to which faculty felt that their workload affected their personal lives. Items for all of these scales were rated on a six-point Likert-type scale ranging from “Strongly Disagree” to “Strongly Agree”, with higher scores indicating greater support and belonging, more inclusive environments, and better work-life balance.

Burnout was measured using a modified five-item (𝛼 = 0.92) instrument [22] derived from the Maslach Burnout Inventory emotional exhaustion subscale [23]. We focused on emotional exhaustion because it is the most reliable indicator of burnout, and this abbreviated measure has been shown to be psychometrically consistent with longer measures [24]. Further, previous research has indicated that emotional exhaustion drives specific demographic differences in the burnout observed in medical educators [7]. Each item was rated on a five-point Likert-type scale ranging from “Strongly Disagree” to “Strongly Agree”, and responses were scaled to range from 0 to 100 with higher scores indicating greater burnout. Job satisfaction and likelihood of recommending the workplace were assessed with scales developed for the purposes of this survey. The job satisfaction scale consisted of three items (𝛼 = 0.72) that assessed overall satisfaction, alignment of values, and job-related stress. Items for the job satisfaction scale were rated on a five-point Likert-type scale ranging from “Strongly Disagree” to “Strongly Agree”, with higher scores indicating greater job satisfaction. Likelihood of recommending the workplace was assessed using a single, eleven-point item ranging from “Not at all likely” to “Extremely likely”. Items for all measures are provided in Appendix A.

### Procedure and Ethical Approval

A designated survey coordinator at each participating campus distributed the online survey via email to faculty in clinical and basic science departments. The median time respondents took to complete the full survey was 25.5 min. The study protocol was reviewed by the WCG Institutional Review Board and was determined to be exempt from full board review.

### Data Analysis

Descriptive statistics were calculated for all measures. Independent samples *t*-tests were used to compare mean scores by gender (male v. female), URiM status (URiM v. non-URiM), and discipline (basic science v. clinical), and Cohen’s *d* was used as a measure of effect size. Cronbach’s alpha was used to estimate the internal reliability of the measures. Bivariate associations among perceived professional development support, workplace belonging, workplace inclusivity, work-life balance, burnout, job satisfaction, and likelihood of recommending the workplace were evaluated using Pearson correlation coefficients. Finally, burnout, job satisfaction, and likelihood of recommending the workplace were separately regressed on gender, URiM status, discipline, and workplace factors: perceived professional development support, workplace belonging, workplace inclusivity, and work-life balance. All quantitative predictors were mean-centered. Given the exploratory nature of the study, statistical significance for all tests was set at a two-tailed alpha level of 0.05. All analyses were performed using the R statistical software (version 4.2.1).

## Results

### Sample

A total of 466 medical educators met our inclusion criteria and were included in the final sample, though an additional 82 participants were excluded from the regression analysis due to missing data on at least one scale. The gender distribution of respondents was 47.2% female and the race/ethnicity distribution was 82.7% non-Hispanic, White, 6.5% Asian, 3.8% Hispanic/Latino, 3.5% two or more races or ethnicities, 3.3% non-Hispanic Black or African American, and 0.3% American Indian or Alaskan Native (with 10.8% of respondents identifying as having a racial or ethnic background that is URiM). Our sample was 59.3% clinical faculty.

### Descriptive Statistics and Bivariate Correlations

The descriptive statistics are presented in Table 1, and bivariate correlations are presented in Table 2. All workplace variables were associated with burnout, job satisfaction, and likelihood of recommending the workplace in their expected direction (e.g., work-life balance was negatively associated with burnout and positively associated with job satisfaction). Further, burnout, job satisfaction, and likelihood of recommending the workplace were all highly correlated, indicating that these outcomes are closely related. Specifically, medical educators experiencing high burnout were more likely to report low job satisfaction and were less likely to recommend their workplace.

**Table 1:** Descriptive statistics and group differences

Variable (Range)	Overall	Gender		URiM Status		Discipline	
	*M* (*SD*)	Female *M* (*SD*)	Male *M* (*SD*)	URiM *M* (*SD*)	Asian/White *M* (*SD*)	Basic Science *M* (*SD*)	Clinical *M* (*SD*)
PD Support (1–6)	4.73 (1.10)	4.68 (1.14)	4.83 (0.98)	4.69 (1.26)	4.75 (1.05)	4.58 (1.13)^*^	4.84 (1.06)^*^
Workplace Belonging (1–6)	4.95 (1.08)	4.91 (1.07)	5.07 (0.99)	4.55 (1.45)^*^	5.05 (0.98)^*^	4.82 (1.11)^*^	5.05 (1.03)^*^
Workplace Inclusivity (1–6)	4.07 (1.12)	3.95 (1.17)^*^	4.23 (0.99)^*^	3.58 (1.22)^*^	4.15 (1.06)^*^	4.01 (1.13)	4.11 (1.11)
Work-life Balance (1–6)	3.85 (1.30)	3.79 (1.28)	4.04 (1.24)	3.62 (1.38)	3.91 (1.26)	3.80 (1.31)	3.90 (1.30)
Burnout (1–5)	2.56 (1.16)	2.64 (1.21)^*^	2.37 (1.05)^*^	2.81 (1.18)	2.51 (1.14)	2.72 (1.18)^*^	2.43 (1.13)^*^
Job Satisfaction (1–5)	3.59 (0.84)	3.57 (0.86)	3.68 (0.77)	3.36 (0.97)^*^	3.65 (0.81)^*^	3.52 (0.89)	3.65 (0.80)
Recommend Workplace (0–11)	7.51 (2.50)	7.40 (2.45)^*^	7.91 (2.20)^*^	7.02 (3.08)	7.7 (2.31)	7.15 (2.67)^*^	7.78 (2.33)^*^

*PD Support* Perceived Professional Development Support, *Recommend Workplace* Likelihood of Recommending the Workplace, Range indicates the possible values for that variable, ^*^ indicates that the group difference was statistically significant at the 0.05 level

**Table 2:** Correlation matrix

	1	2	3	4	5	6	7
1. PD Support	1						
2. Workplace Belonging	0.60	1					
3. Workplace Inclusivity	0.41	0.49	1				
4. Work-life Balance	0.41	0.40	0.38	1			
5. Burnout	− 0.48	− 0.59	− 0.53	− 0.77	1		
6. Job Satisfaction	0.57	0.76	0.49	0.60	− 0.75	1	
7. Recommend Workplace	0.61	0.78	0.49	0.47	− 0.68	0.75	1

*PD Support* Perceived Professional Development Support, *Recommend Workplace* Likelihood of Recommending the Workplace, All correlations were statistically significant at the 0.001 level

### Predicting Burnout, Job Satisfaction, and Likelihood to Recommend Their Workplace

The regression model coefficients are presented in Table 3. Overall, the models accounted for 68.5% of the variance in burnout [*F* (7, 376) = 116.66, *p* <.001], 69.2% of the variance in job satisfaction [*F* (7, 376) = 120.53, *p* <.001], and 63.9% of the variance in the likelihood to recommend the workplace [*F* (7, 376) = 95.03, *p* <.001]. Workplace belonging, workplace inclusivity, and work-life balance were significant predictors in all models and perceived professional development support was a significant predictor of likelihood of recommending the workplace. Workplace belonging was the strongest predictor of job satisfaction and likelihood of recommending the workplace, and was also a strong predictor of burnout, indicating a central role as a workplace factor. Similarly, work-life balance was the strongest predictor of burnout and a strong predictor of job satisfaction and likelihood of recommending the workplace.

**Table 3:** Regression model coefficients

	Burnout			Job Satisfaction			Recommend Workplace		
Term	$\:\beta\:$	*b* (95% CI)	*p*	$\:\beta\:$	*b* (95% CI)	*p*	$\:\beta\:$	*b* (95% CI)	*p*
Intercept	0.00	2.45 (2.23, 2.67)	< 0.001	0.00	3.61 (3.46, 3.77)	< 0.001	0.00	7.78 (7.30, 8.26)	< 0.001
Male	−0.03	−0.08 (−0.21, 0.05)	0.238	−0.01	−0.02 (−0.11, 0.08)	0.700	0.03	0.15 (−0.14, 0.43)	0.322
Asian or White	0.03	0.11 (−0.10, 0.32)	0.314	−0.01	−0.03 (−0.19, 0.12)	0.676	−0.03	−0.22 (−0.68, 0.25)	0.364
Basic Science	0.07	0.15 (0.02, 0.29)	0.024	0.02	0.03 (−0.07, 0.13)	0.535	−0.05	−0.23 (−0.52, 0.06)	0.120
Perceived Professional Development Support	−0.02	−0.03 (−0.10, 0.05)	0.522	0.05	0.04 (−0.02, 0.09)	0.193	0.15	0.33 (0.16, 0.50)	< 0.001
Workplace Belonging	−0.22	−0.24 (−0.33, −0.16)	< 0.001	0.55	0.44 (0.38, 0.51)	< 0.001	0.55	1.26 (1.07, 1.44)	< 0.001
Workplace Inclusivity	−0.20	−0.21 (−0.28, −0.14)	< 0.001	0.08	0.06 (0.01, 0.11)	0.021	0.13	0.28 (0.12, 0.44)	< 0.001
Work-life balance	−0.57	−0.52 (−0.57, −0.46)	< 0.001	0.35	0.23 (0.19, 0.27)	< 0.001	0.13	0.24 (0.11, 0.36)	< 0.001
Model Fit									
R ^2^	0.685			0.692			0.639		
RMSE	0.645			0.464			1.416		

*β * standardized beta coefficient, *b* unstandardized beta coefficient, 95% *CI* the lower and upper bounds of the 95% confidence intervals, *RMSE* root mean square error

### Group Differences

Compared to males, female faculty reported significantly less workplace inclusivity [*t* (410) = −2.63, *p* =.009, *d* = −0.26], more burnout [*t* (412) = 2.40, *p* =.017, *d* = 0.24], and were less likely to recommend their workplace [*t* (412) = −2.25, *p* =.025, *d* = −0.22]. Compared to their White or Asian peers, URiM faculty reported significantly less belonging [*t* (396) = −2.98, *p* =.003, *d* = −0.49], less workplace inclusivity [*t* (394) = −3.26, *p* =.001, *d* = −0.53], and lower job satisfaction [*t* (396) = −2.16, *p* =.031, *d* = −0.35]. Finally, compared to clinical faculty, basic science faculty reported significantly less professional development support [*t* (436) = −2.44, *p* =.015, *d* = −0.24], less belonging [*t* (453) = −2.24, *p* =.026, *d* = −0.21], more burnout [*t* (458) = 2.62, *p* =.009, *d* = 0.25], and were less likely to recommend their workplace [*t* (452) = −2.66, *p* =.008, *d* = −0.25]. 

## Discussion

In the present study, we collected data from faculty at 42 medical school campuses across the nation, allowing us to obtain a large and diverse sample. Significant differences related to gender, race/ethnicity, and academic discipline indicate that the workplace is not experienced the same by all educators, with female, URiM and basic science faculty reporting lower outcomes than their peers. This indicates that interventions to support medical educator well-being should be targeted to the specific needs of different faculty. Medical schools must recognize and address these unique challenges by fostering more inclusive and supportive environments.

The regression models indicate that personal identities and workplace factors explain a significant amount of variance in burnout, job satisfaction, and likelihood of recommending their workplace. Workplace belonging and work-life balance were particularly strong predictors across the three predictors, indicating a central role in supporting medical educators in the workplace. Given that basic science faculty and educators who are URiM showed less belonging than their peers, it is particularly important to support these groups. Additionally, these educators tend to serve as important mentors to first year medical students (who are taking more basic science courses) and students from similar racial or ethnic backgrounds, highlighting the importance of supporting medical educator well-being.

Our findings also indicated important gaps in the experiences of females and medical educators part of an underrepresented racial or ethnic group. Compared to their male colleagues, female educators reported feeling like the workplace was less inclusive, experienced more burnout, and were less likely to recommend their workplace. This finding is consistent with what others have observed in academic medicine [5, 6] and in clinical settings [7]. Compared to their Asian or White colleagues, medical educators who are part of an underrepresented racial or ethnic group reported less belonging, a less inclusive workplace, and lower job satisfaction, which is also consistent with the literature [9]. These findings suggest that addressing gender and racial/ethnic disparities can help institutions build more supportive and inclusive workplaces, and, consequently, retain a more diverse faculty. A goal of future research should be to identify the specific mechanism(s) that create these inequitable experiences (e.g., unequal workloads or expectations, experiences of racial or gender discrimination), so interventions can be more targeted for specific workplaces.

The significance of these findings is especially relevant in today’s complex political and financial climate, which presents increasingly challenging conditions for securing and maintaining careers in medicine. Historically, the science, technology, engineering, and mathematics (STEM) pipeline has enabled educators to serve as invaluable role models and mentors for students aspiring to careers in STEM. However, the growing prevalence of educator burnout is likely to discourage students, who may come to view a future in these fields as overly stressful, unstable, or demanding. Consequently, a weakened support system for URiM students could result in fewer applications from this group. Additionally, female and basic science educators, who are more likely to report burnout and less likely to recommend their workplace, face similar challenges, further impacting the STEM pipeline for students with these underrepresented personal identities. As a result, students risk losing access to diverse educators and may encounter diminished opportunities for mentorship and career advancement.

Despite obtaining a large and diverse sample, there were still limitations to our survey approach. The survey contained numerous items for faculty members to answer, which likely led to lower completion rates. Relatedly, we may have a biased sample because faculty who are burned out may have been more likely to want to report their feelings or less likely because the length of the survey felt burdensome. Additionally, due to the survey administration method, it is unclear how many faculty members had the opportunity to participate, making it impossible to calculate accurate response rates. Another limitation is that our data reflect faculty attitudes from one time point, and we cannot conclude what trends have occurred over time. For future studies, we suggest shortening the survey to respect faculty time and reduce survey fatigue, while also making it more feasible to conduct the survey annually to track faculty well-being. Only by collecting this data over time can institutions track progress and evaluate initiatives designed to reduce burnout and increase job satisfaction.

Our findings suggest that institutions can take targeted action to foster a healthy and supportive workplace, which in turn may lower turnover and increase faculty well-being. Medical school programs looking to improve their workplace may want to try mentorship programs. For example, a small group faculty development program at the Vanderbilt University School of Medicine was created to provide mentorship, support time management, and develop leadership, among other skills [25]. This program resulted in increased professional development support, scholarly activity, and connections among faculty, including those not in the program. The results were most pronounced for female faculty, who, in our data, are most likely to experience burnout. This program was designed by senior faculty to support junior faculty, which meant it was better able to respond to the local pressures on that specific campus. More generally, allocating protected time for teaching and research, reducing administrative tasks, establishing mentorship programs to support junior faculty in developing work-life balance and increase belonging, and providing leadership training that emphasizes transparency and support can strengthen the campus climate. By providing both structural supports (e.g., mentorship programs, protected time) and professional development support, medical school administrators have the ability to impact faculty well-being.

## References

[1] Lapinski J, Yost M, Sexton P, et al. Factors modifying burnout in osteopathic medical students. Acad Psychiatry. 2016;40(1):55–62. 10.1007/s40596-015-0375-0. 26108394 10.1007/s40596-015-0375-0

[2] Ley AF, Han JJ, Hare E, Sikorshii A, Taylor JR, Shahed A, Guro C. Beyond burnout: a four-year survey of osteopathic medical student mental health and the implications for the development of wellness and mental health programs. J Osteopath Med. 2023;123(5):225–33. 10.1515/jom-2022-0179. 36825542 10.1515/jom-2022-0179

[3] Himmelberger ZM, Tibbetts Y, Barron KE, Hulleman CS, Harootunian G, Speicher MR. How can a growth mindset-supportive learning environment in medical school promote student well-being? Fam Syst Health. 2024;42(3):343–54. 10.1037/fsh0000915. 39418421 10.1037/fsh0000915

[4] Schloss EP, Flanagan DM, Culler CL, Wright AL. Some hidden costs of faculty turnover in clinical departments in one academic medical center. Acad Med. 2009;84(1):32–6. 10.1097/ACM.0b013e3181906dff. 19116474 10.1097/ACM.0b013e3181906dff

[5] Silver EM, Balas K, Ellinas EH, Jacobs JW, Booth GS, Dandar VM, Silver JK. Sense of belonging and intent to leave among medical school faculty. JAMA Netw Open. 2025;8(4):e257728. 10.1001/jamanetworkopen.2025.7728. 40266621 10.1001/jamanetworkopen.2025.7728 PMC12019519

[6] Pololi LH, Civian JT, Brennan RT, Dottolo AL, Krupat E. Experiencing the culture of academic medicine: gender matters, a National study. J Gen Intern Med. 2013;28(2):201–7. 10.1007/s11606-012-2207-1. 22936291 10.1007/s11606-012-2207-1 PMC3614142

[7] Lyubarova R, Salman L, Rittenberg E. Gender differences in physician burnout: driving factors and potential solutions. Perm J. 2023;27(2):130–6. 10.7812/TPP/23.023. 37303223 10.7812/TPP/23.023 PMC10266850

[8] Artz B, Kaya I, Kaya O. Gender role perspectives and job burnout. Rev Econ Househ. 2022;20(2):447–70. 10.1007/s11150-021-09579-2. 34429716 10.1007/s11150-021-09579-2 PMC8375289

[9] Pololi LH, Evans AT, Gibbs BK. The experience of minority faculty who are underrepresented in medicine, at 26 representative U.S. medical schools. Acad Med. 2013;88(9):1308–14. 10.1097/ACM.0b013e31829ed0be. 23887015 10.1097/ACM.0b013e31829eefff

[10] Pololi L, Cooper LA, Carr P. Race, disadvantage and faculty experiences in academic medicine. J Gen Intern Med. 2010;25(12):1363–9. 10.1007/s11606-010-1478-7. 20697960 10.1007/s11606-010-1478-7 PMC2988158

[11] Shanafelt TD, Boone S, Tan L, et al. Burnout and satisfaction with work-life balance among US physicians relative to the general US population. Arch Intern Med. 2012;172(18):1377–85. 10.1001/archinternmed.2012.3199. 22911330 10.1001/archinternmed.2012.3199

[12] Shanafelt TD, Hasan O, Dyrbye LN, et al. Changes in burnout and satisfaction with work-life balance in physicians and the general US working population between 2011 and 2014. Mayo Clin Proc. 2015;90(12):1600–13. 10.1016/j.mayocp.2015.08.023. 26653297 10.1016/j.mayocp.2015.08.023

[13] Dandar VM, Bunton SA, Grigsby RK. Burnout among faculty physicians in U.S. medical schools: prevalence and prevention. Poster presented at: The University of Texas System Conference: Beyond Resiliency Training: Organizational Strategies to Alleviate Burnout and Increase Wellness in Academic Medicine; September 25–26, 2017; Houston, TX.

[14] Dandar VM, Grigsby RK, Bunton SA. Burnout among U.S. medical school faculty. Anal Brief. 2019;19(1):1–3.

[15] Haghighinejad H, Jafari P, Rezaie M, Farrokhi M, Jafari M, Ramzi M. Burnout comparison between clinical and basic sciences faculty of a medical school and evaluation of related factors. Iran J Psychiatry. 2021;16(4):399–408. 10.18502/ijps.v16i4.7227. 35082852 10.18502/ijps.v16i4.7227 PMC8725187

[16] Shiri R, El-Metwally A, Sallinen M, Pöyry M, Härmä M, Toppinen-Tanner S. The role of continuing professional training or development in maintaining current employment: a systematic review. Healthcare. 2023;11(21):2900. 10.3390/healthcare11212900. 37958044 10.3390/healthcare11212900 PMC10647344

[17] Cross W, Wyman PA. Training and motivational factors as predictors of job satisfaction and anticipated job retention among implementers of a school-based prevention program. J Prim Prev. 2006;27(2):195–215. 10.1007/s10935-005-0018-4. 16550457 10.1007/s10935-005-0018-4

[18] Tibbetts Y, Himmelberger ZM, Barron KE, Speicher MR, Hulleman CS. Learning mindsets and well-being and ill-being among osteopathic medical students. JAMA Netw Open. 2024;7(6):e2418090. 10.1001/jamanetworkopen.2024.18090. 38874920 10.1001/jamanetworkopen.2024.18090 PMC11179131

[19] Kim G, Yu H, Ryu E. Social group membership, burnout, and subjective well-being in new nurses in the life transition period: a cross-sectional study. Nurs Open. 2023;10(5):3295–304. 10.1002/nop2.1581. 36575584 10.1002/nop2.1581 PMC10077367

[20] Skaalvik EM, Skaalvik S. Shared goals and values in the teaching profession, job satisfaction and motivation to leave the teaching profession: the mediating role of psychological need satisfaction. Soc Psychol Educ. 2023;26:1227–44. 10.1007/s11218-023-09787-x.

[21] Duke N, Gross A, Moran A, et al. Institutional factors associated with burnout among assistant professors. Teach Learn Med. 2019;10. 10.1080/10401334.2019.1638263. 10.1080/10401334.2019.1638263 31315454

[22] Sexton JB, Adair KC, Leonard MW, et al. Providing feedback following leadership walkrounds is associated with better patient safety culture, higher employee engagement and lower burnout. BMJ Qual Saf. 2018;27:261–70. 10.1136/bmjqs-2016-006399. 10.1136/bmjqs-2016-006399 PMC5867443 28993441

[23] Maslach C, Jackson SE. The measurement of experienced burnout. J Organ Behav. 1981;2(2):99–113. 10.1002/job.4030020205.

[24] Penny CL, Adair KC, Frankel AS, et al. A new look at an old well-being construct: evaluating the psychometric properties of 9-, 5-, and 1-item versions of emotional exhaustion metrics. Front Psychol. 2023;14:1267660. 10.3389/fpsyg.2023.1267660. 38078261 10.3389/fpsyg.2023.1267660 PMC10702494

[25] Fleming GM, Simmons JH, Xu M, et al. A facilitated peer mentoring program for junior faculty to promote professional development and peer networking. Acad Med. 2015;90(6):819–26. 10.1097/ACM.0000000000000705. 25830537 10.1097/ACM.0000000000000705 PMC4446138

